# Characterization of white matter alterations using diffusion kurtosis imaging in patients with amyotrophic lateral sclerosis

**DOI:** 10.1002/brb3.3102

**Published:** 2023-06-06

**Authors:** Tanushka Anand, Abdullah Ishaque, Daniel Ta, Muhammad Umer Khan, Komal Bharti, Andrew Wu, Dennell Krebs, Christian Beaulieu, Peter Seres, Sanjay Kalra

**Affiliations:** ^1^ Division of Neurology, Department of Medicine University of Alberta Edmonton Canada; ^2^ Neuroscience and Mental Health Institute University of Alberta Edmonton Canada; ^3^ Faculty of Medicine and Dentistry University of Alberta Edmonton Canada; ^4^ Department of Computing Sciences University of Alberta Edmonton Canada; ^5^ Department of Biomedical Engineering University of Alberta Edmonton Canada

**Keywords:** corpus callosum, corticospinal tract, diffusion kurtosis imaging, diffusion tensor imaging, upper motor neuron

## Abstract

**Background:**

To evaluate the degeneration of the corticospinal tract (CST) and corpus callosum (CC) in patients with motor neuron disease and upper motor neuron (UMN) dysfunction using diffusion kurtosis imaging (DKI).

**Methods:**

Twenty‐seven patients and 33 healthy controls underwent magnetic resonance imaging along with clinical and neuropsychological testing. Tractography of diffusion tensor images was performed to extract tracts of the bilateral CST and CC. Group mean differences both across the entire averaged tract and along each tract were assessed, including correlations between diffusion metrics and clinical measures. Tract‐based spatial statistics (TBSS) was performed to evaluate the spatial distribution of whole‐brain microstructural abnormalities in patients.

**Results:**

In comparison to controls, patients had significantly higher mean and radial diffusivity and lower fractional anisotropy (FA), kurtosis anisotropy, mean kurtosis (MK), and radial kurtosis (RK) in the CST and CC (*p* < .017). Along‐the‐tract analysis revealed changes concentrated in the posterior limb of the internal capsule, corona radiata, and primary motor cortex (false‐discovery rate *p* < .05). FA of the left CST correlated with disease progression rate, whereas MK of the bilateral CST correlated with UMN burden (*p* < .01). TBSS results corroborated along‐tract analysis findings and additionally revealed reduced RK and MK in the fornix, where diffusion tensor imaging (DTI) changes were absent.

**Conclusion:**

DKI abnormalities in the CST and CC are present in patients with UMN dysfunction, potentially revealing complementary information to DTI regarding the pathology and microstructural alterations occurring in such patients. DKI shows promise as a potential in vivo biomarker for cerebral degeneration in amyotrophic lateral sclerosis.

## Graphical abstract

Evaluation of the degeneration of the corpus callosum in patients with upper motor neuron disease using diffusion tensor and kurtosis imaging. Findings from along‐tract analysis and TBSS suggest that DKI might be a useful tool in evaluating cerebral degeneration.

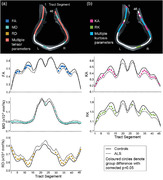

## 1 INTRODUCTION

Motor neuron diseases (MND) are characterized by progressive degeneration of the human motor system resulting in the loss of motor control. Amyotrophic lateral sclerosis (ALS) and primary lateral sclerosis (PLS) are types of MND that present with upper motor neuron (UMN) degeneration. However, ALS is also associated with the concurrent deterioration of both UMN and lower motor neurons (LMN), whereas only UMNs are affected in PLS. Although LMN dysfunction is detected based on electromyography results and nerve conduction studies, there is currently no biomarker that detects in vivo cerebral degeneration. Therefore, the diagnosis of UMN disease is obtained indirectly through clinical neurological examinations, resulting in significant diagnostic uncertainty (Woo et al., 2014).

Diffusion tensor imaging (DTI), a robust modality of magnetic resonance imaging (MRI), can detect white matter (WM) alterations based on the principle of Gaussian approximation of diffusion displacement of water molecules (Steven et al., 2014). DTI metrics, such as fractional anisotropy (FA), an indirect measure of fiber directionality and axonal loss (Sbardella et al., 2013), has potential as a biomarker for UMN dysfunction (Caiazzo et al., 2014). However, the complex intracellular and extracellular in vivo environment causes the diffusion of water molecules to deviate considerably from a Gaussian distribution.

As an extension of DTI, diffusion kurtosis imaging (DKI), a novel method that considers the non‐Gaussian diffusion of water molecules in tissue, detects microstructural alterations in WM regions with complex arrangements, such as crossing fibers (Fieremans et al., 2011, Jensen & Helpern, 2010). Additionally, DKI can detect changes in isotropic structures, such as gray matter thereby allowing whole‐brain microstructural analyses for various neurological disorders (Jensen et al., 2005, Lu et al., 2006). DKI is measured using metrics such as kurtosis anisotropy (KA); analogous to FA, mean kurtosis (MK); an average of diffusion kurtosis along all directions, axial kurtosis (AK), and radial kurtosis (RK); measures of tissue heterogeneity axial and perpendicular to the direction maximal diffusion, respectively (Andica, 2020). DKI has been successfully employed to understand the pathogenesis of neurological disorders, such as Alzheimer's (Praet et al., 2018), Parkinson's (Sejnoha et al., 2020), multiple sclerosis (Li et al., 2018), and epilepsy (Winston, 2015).

Conventional tractography methods average diffusion metrics across multiple tract voxels, yielding a single mean value, resulting in the loss of regional information. Along‐tract analysis provides a more comprehensive understanding of the variation in diffusion metrics throughout the tracts of interest (Colby et al., 2012). Along‐tract analysis of DTI metrics of the corticospinal tract (CST) in ALS shows decreased FA at the internal capsule (Ishaque et al., 2019, Sarica et al., 2014, Sarica et al., 2017). However, along‐tract analysis of DKI metrics in UMN diseases, such as ALS, is yet to be evaluated. This study aimed to evaluate the degeneration of the CST and corpus callosum (CC) in patients with MND and UMN dysfunction using DKI. It was hypothesized that DKI metrics in the CST and CC will be abnormal. DKI findings in the CST and CC will correlate with clinical measures of UMN dysfunction and disease progression rate, and DKI will be more robust in the detection of UMN dysfunction than DTI.

## 2 METHODS

### 2.1 Participants

For this study, 27 patients (26 ALS and 1 PLS) and 33 controls were recruited through the Canadian ALS Neuroimaging Consortium (CALSNIC) from the University of Alberta. To be included in the present study, patients were required to present evidence of clinical UMN signs on neurological examination. Furthermore, a diagnosis of possible, probable lab supported, probable or definite ALS as defined by the El Escorial criteria (Brooks et al., 2000) or PLS was required for inclusion. Patients were excluded if they presented with comorbid neurological disorders. Controls without a history of psychiatric or neurological disease were recruited. Data collection for this study was approved by the University of Alberta Research Ethics Board, and all participants provided informed written consent.

Clinical evaluations were performed on the day of the MRI scan. The ALS Functional Rating Scale‐Revised (ALSFRS‐R) was collected to measure disease severity, ranging from 0 to 48. One patient did not complete the ALSFRS‐R and was excluded from related analyses. Disease progression rate was calculated; 48—ALSFRS‐R score/symptom duration in months. Finger and foot‐tapping scores were calculated by recording the average number of taps over 10 seconds using two trials for left and right sides. The degree of UMN dysfunction was calculated based on the presence of spasticity and hyperreflexia in the upper and lower extremities. UMN burden was assessed out of a maximum score of 6 points for each side (Ta et al., 2021). Participant demographic and clinical scores are provided in Table 1.

**TABLE 1:** Study participant characteristics. Values are shown as mean ± standard deviation unless otherwise stated.

Characteristics	Controls	Patients	*p*‐Values
*n*	33	27	
Age, years	54.1 ± 10.4	58.8 ± 12.2	.1^a^
Gender (male, female)	17,16	13,14	.7^b^
Symptom onset (bulbar, limb)	–	21, 78	–
ALSFRS‐R^c^	–	37.8 ± 6.7	–
Symptom duration, months	–	24.7 ± 14.6	–
Disease progression rate^c^	–	0.51 ± 0.39	–
UMN burden	–	Right = 2.4 ± 1.7	
		Left = 2.4 ± 1.6	
Finger tapping	Right = 67.4 ± 11.9	Right = 42.8 ± 17.1	Right (<.001)^a^
	Left = 58.0 ± 9.9	Left = 40.1 ± 14.9	Left (<.001)^a^
Foot tapping	Right = 42.2 ± 9.8	Right = 26.6 ± 13.1	Right (<.001)^a^
	Left = 41.1 ± 9.7	Left = 25.3 ± 11.4	Left (<.001)^a^

Abbreviations: ALSFRS‐R, ALS Functional Rating Scale‐Revised; UMN, upper motor neuron.

^a^ Two‐sample independent *t*‐test.

^b^ Chi‐squared test.

^c^ One patient was excluded because of missing ALSFRS‐R score.

### 2.2 Data acquisition

Participants were scanned in a Siemens Prisma 3T scanner at the Peter S Allen MRI Research Centre at the University of Alberta. Diffusion‐weighted images were acquired axially with a two‐dimensional spin echo, single‐shot echo‐planar imaging (EPI) pulse sequence with the following scan parameters: slice thickness = 2 mm, field of view = 256 × 256 mm^2^, matrix dimensions = 256 × 256, voxel size = 1 × 1 × 2 mm^3^ (interpolated from 2 × 2 × 2 mm^3^), TR = 8000 ms, TE = 60 ms, flip angle = 90°, number of slices = 70, b0 = 5, *b* values = 0, 1000, 2000 s/mm^2^ for 30 directions was used. T1W images were acquired axially with a 3D MPRAGE sequence (slice thickness = 1 mm, voxel size = 1 × 1 × 1 mm^3^, TR = 1600 ms, TE = 3.8 s, flip angle = 15°, number of slices = 128).

### 2.3 Data processing

DTI and DKI data were preprocessed using ExploreDTI version 4.8.6 (Leemans et al.). Signal drift, subject motion, and eddy current corrections were performed using ExploreDTI (Perrone et al., 2015, Vos et al., 2017). A deterministic fiber tracking approach was performed for whole‐brain tractography with the following parameters: FA threshold = 0.2, fiber length range = 50–500 mm, and angle threshold = 30° (Ishaque et al., 2019). Regions of interest (ROI) were manually placed to extract the CST and motor callosal tracts. Specifically, the segmentation of the left CST was performed using a “SEED” ROI in the left pons and an “AND” ROI on the left precentral gyrus. The same procedure was used to reconstruct the right CST. The “AND” ROI was placed on the left and right precentral gyrus to extract tracts protruding from the body of the CC into the primary motor cortex. Anatomically inaccurate tracts were excluded using “NOT” ROIs. Intra‐rater reliability was tested by manually tracking all subjects twice, blinded to subject number. FA, MK, and absolute tract volume of each tract were chosen to assess intra‐rater reliability.

### 2.4 Group level and along‐tract analysis

Along‐tract analysis of DTI and DKI metrics was performed on the right CST, left CST, and CC, by dividing each tract into 46 discrete points, computing group differences at each point. Outliers due to signal loss in lower regions of the tracts were eliminated. Group level comparisons were performed by averaging diffusion and kurtosis metrics along the tract to obtain the mean value for each metric.

### 2.5 Tract‐based spatial statistics (TBSS)

Tract‐based spatial statistics (TBSS) of DTI and DKI data was implemented on the FMRIB Software Library 4.1.6 (FSL, Oxford Centre for Functional MRI of the Brain, UK) (www.fmrib.ox.ac.uk/fsl). DTI and DKI maps were registered to T1‐weighted images and corrected for motion and EPI distortion using ExploreDTI and then processed in FSL. Participant FA maps were aligned to standard Montreal Neurological Institute (MNI152) space, after which the mean FA image and mean FA skeleton were generated. The mean FA maps were thresholded at 0.2 to include only WM of major tracts and to exclude any gray matter.

### 2.6 Statistics

Statistical analyses were performed using IBM SPSS for Windows Version 24.0. Intraclass correlation coefficient (ICC) values of FA, MK, and tract volume were used to assess intra‐rater reliability of tracking methods using a two—way mixed model with a 95% confidence interval. Independent *t*‐tests were used to assess demographic differences between groups. Differences in gender proportions were assessed using chi‐squared tests. Shapiro–Wilk tests were conducted to test for normality for DTI and DKI metrics extracted from all tracts. Group comparisons of DTI and DKI metrics were examined using one‐way analysis of covariance with age and gender as nuisance variables. After correcting for multiple comparisons *p* < .05/3, the level of statistical significance was set at *p* < .017. Effect sizes were calculated and expressed as partial *η* ^2^.

Independent *t*‐tests were performed for along‐tract analysis of the CST and CC to analyze group comparisons at each point. Threshold for statistical significance was set at false‐discovery rate (FDR)‐corrected *p* < .05. Pearson correlations coefficients were used to determine linear associations with the clinical measures; disease progression rate, the ALSFRS‐R, contralateral finger and foot‐tapping scores, and UMN burden. After correcting for multiple comparisons (*p* < .05/5), statistical significance for correlation analysis was accepted at *p* < .01.

Age and gender were considered covariates for TBSS analysis. Voxel‐wise between group statistics were performed after projecting each participant's aligned FA image to the mean FA skeleton. Next, tensor and kurtosis metric maps were also projected onto the mean FA maps to conduct voxel‐wise statistics. A generalized linear model with appropriate design and contrast matrices was utilized for group comparisons for all diffusivity metrics. All results shown were significant at the cluster level (threshold‐free cluster enhancement [TFCE]) and thresholded at *p* < .05. Results were also presented if significance was obtained with a FDR corrected threshold of *p* < .05. WM tracts containing clusters with significant group differences were identified using the John Hopkins University (JHU) WM tractography atlas and international consortium for brain mapping‐DTI‐81 WM labels atlas (Hua et al., 2008, Wakana et al., 2007).

## 3 RESULTS

### 3.1 Demographics

Sixty participants were included in this study (27 patients, 33 controls). No significant difference in age or gender between groups was present. For patients, the mean ALSFRS‐R ± standard deviation was 37.8 ± 6.68. The mean ± standard deviation and median symptom duration were 24.7 ± 14.6 months and 21.06, respectively. The mean ± standard deviation and median disease progression rate were 0.51 ± 0.39 and 0.34 per month. A summary of participant demographics is provided in Table 1.

### 3.2 Whole‐tract group differences in DTI and DKI metrics

ICC results showed moderate‐to‐excellent reproducibility. Shapiro–wilk test of DKI and DTI metrics for all tracts revealed normal distribution of values (*p* > .05); thereby, parametric tests were used to test for statistical significance. Seven participants (6 patients, 1 control) from WM tract analyses of the CC and one participant for the right CST (1 patient) analysis were excluded due to poor tract quality. Removal of the PLS patient in our group analysis did not reveal any changes in the significance of our results and was therefore included in our overall analysis. For DTI analysis, RD of the LCST (*η*_p_^2^ = .246) and RCST (*η*_p_^2^ = .229) showed the most pronounced group differences, and in the CC, MD (*η*_p_^2^ = .236) was altered to a larger extent. However, in DKI analysis, significantly lower KA (*η*_p_^2^ = .176) was obtained in the LCST. For the RCST and CC, most significant group differences were also found in KA *η*_p_^2^ = .122, *η*_p_^2^ = .189, respectively. Group differences in DTI and DKI measures are provided in Tables 2 and 3, respectively.

**TABLE 2:** Group differences in the mean diffusion tensor imaging (DTI) metrics of corpus callosum and corticospinal tract between controls and patients.

	Controls	Patients	*p* Value	Effect size (*η*_p_^2^)	*df*	*F* Value
***Left corticospinal tract***						
FA	0.56 ± 0.03	0.53 ± 0.03	**.001**	.18	1	12.47
MD (×10^−3^ mm^2^/s)	0.86 ± 0.03	0.89 ± 0.03	**.0004**	.20	1	13.92
AD (×10^−3^ mm^2^/s)	1.48 ± 0.05	1.50 ± 0.06	.57	.01	1	0.32
RD (×10^−3^ mm^2^/s)	0.55 ± 0.03	0.59 ± 0.03	**.000008**	.25	1	18.25
***Right corticospinal tract***						
FA	0.55 ± 0.03	0.52 ± 0.03	**.001**	.19	1	12.86
MD (×10^−3^ mm^2^/s)	0.88 ± 0.03	0.92 ± 0.03	**.001**	.19	1	13.22
AD (×10^−3^ mm^2^/s)	1.51 ± 0.04	1.51 ± 0.05	1.00	.0	1	0.00
RD (×10^−3^ mm^2^/s)	0.58 ± 0.04	0.62 ± 0.04	**.0002**	.23	1	16.35
***Corpus callosum***						
FA	0.51 ± 0.03	0.48 ± 0.04	**.001**	.20	1	12.38
MD (×10^−3^ mm^2^/s)	0.98 ± 0.04	1.04 ± 0.05	**.0003**	.24	1	15.13
AD (×10^−3^ mm^2^/s)	1.62 ± 0.05	1.65 ± 0.05	.04	.08	1	4.39
RD (×10^−3^ mm^2^/s)	0.67 ± 0.05	0.72 ± 0.06	**.001**	.22	1	13.57

*Note*: Analyses of covariance tests were used to detect the differences between groups after controlling for age and gender. Statistical threshold was set at *p* < .017.

Abbreviations: AD, axial diffusivity; *df*, degrees of freedom; FA, fractional anisotropy; MD, mean diffusivity; RD, radial diffusivity.

**TABLE 3:** Group differences in the mean diffusion kurtosis imaging (DKI) metrics of corpus callosum and corticospinal tract between controls and patients.

	Controls	Patients	*p* Value	Effect size (*η*_p_^2^)	*df*	F value
*Left corticospinal tract*						
KA	0.55 ± 0.06	0.49 ± 0.06	**.001**	.18	1	11.93
MK	1.17 ± 0.11	1.11 ± 0.11	**.008**	.12	1	7.61
AK	0.75 ± 0.04	0.74 ± 0.04	.18	.03	1	1.85
RK	1.60 ± 0.23	1.46 ± 0.23	**.009**	.12	1	7.38
*Right corticospinal tract*						
KA	0.50 ± 0.05	0.46 ± 0.06	**.008**	.12	1	7.67
MK	1.14 ± 0.09	1.10 ± 0.10	.04	.07	1	4.23
AK	0.74 ± 0.03	0.74 ± 0.04	.58	.01	1	0.31
RK	1.55 ± 0.19	1.47 ± 0.21	.09	.05	1	3.04
*Corpus callosum*						
KA	0.43 ± 0.05	0.38 ± 0.06	**.001**	.19	1	11.41
MK	1.05 ± 0.10	1.00 ± 0.09	.03	.09	1	4.83
AK	0.75 ± 0.04	0.74 ± 0.04	.4	.02	1	0.78
RK	1.59 ± 0.20	1.44 ± 0.20	**.008**	.13	1	7.59

*Note*: Analyses of covariance tests were used to detect the differences between groups after controlling for age and gender. Statistical threshold was set at *p* < .017.

Abbreviations: AK, axial kurtosis; *df*, degrees of freedom; KA, kurtosis anisotropy; MK, mean kurtosis; RK, radial kurtosis.

### 3.3 Correlations of DTI and DKI metrics with clinical scores of disease progression and UMN burden (Figure 1)

Disease progression rate was significantly negatively correlated with FA of the LCST (*r* = −.564, *p* = .003). MK of the left CST (*r* = −.679, *p =* .001) and right CST (*r* = −.541, *p =* .005) was negatively correlated with contralateral UMN scores, respectively.

### 3.4 Along‐tract analysis of corticospinal tract and corpus callosum

Significant group differences in DTI metrics (FDR corrected, *p* < .05) were observed in the left and right CST (Figure 2). Specifically, along‐tract analyses revealed a significantly higher MD in the left and right CST extending from the internal capsule to the motor cortex in patients, whereas higher RD was observed throughout the tract in patients compared to controls. Significantly lower FA was observed in the WM tracts of the left CST projecting into the motor cortex, whereas in the right CST, reduced FA was observed at the internal capsule in patients relative to controls. Furthermore, significantly lower MK was observed in the left CST from the internal capsule to the motor cortex in patients. Reductions in RK in patients were observed in the left CST at the level of the centrum semiovale and primary motor cortex. KA in patients was reduced in regions of the left CST extending from the internal capsule to the motor cortex, whereas in the right CST similar patterns of differences in diffusion tensor and kurtosis metrics were obtained at the level of the internal capsule and motor cortex only. Along‐tract analyses in the CC revealed significantly lower FA, KA, RK and higher MD and RD metrics in patients compared to controls, with more differences clustered at the ends of the callosal fibers projecting into the motor cortex than in the central regions (Figure 3 ). No significant group differences were obtained for AD or AK.

**FIGURE 1:**
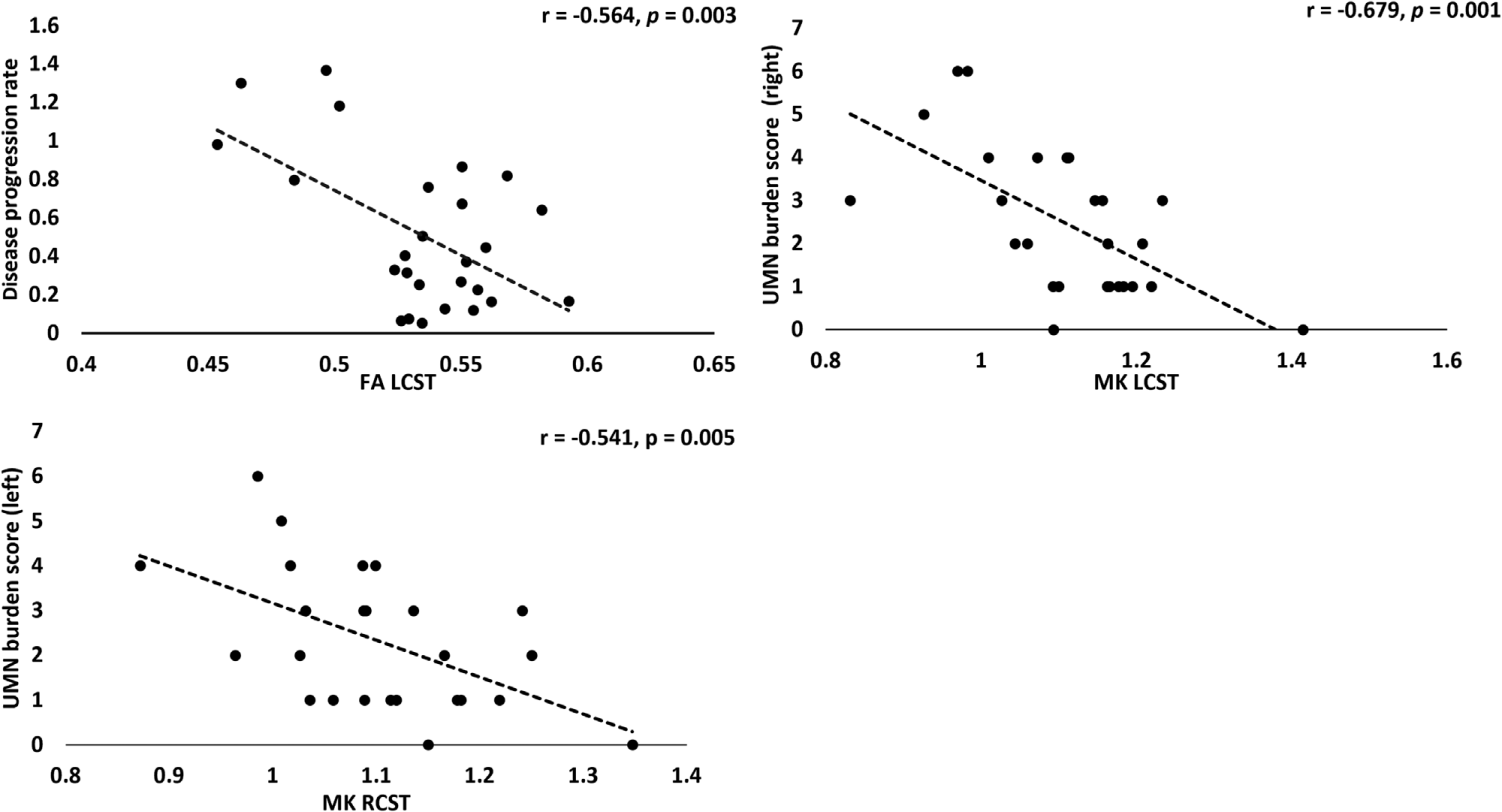
Correlations were observed between diffusion tensor imaging (DTI) and diffusion kurtosis imaging (DKI) measures in the motor fibers of the left corticospinal tract and right corticospinal tract with clinical scores such as disease progression rate and upper motor neuron (UMN) burden scores. Threshold for significance was set at *p* < .01. FA, fractional anisotropy, MK, mean kurtosis.

**FIGURE 2:**
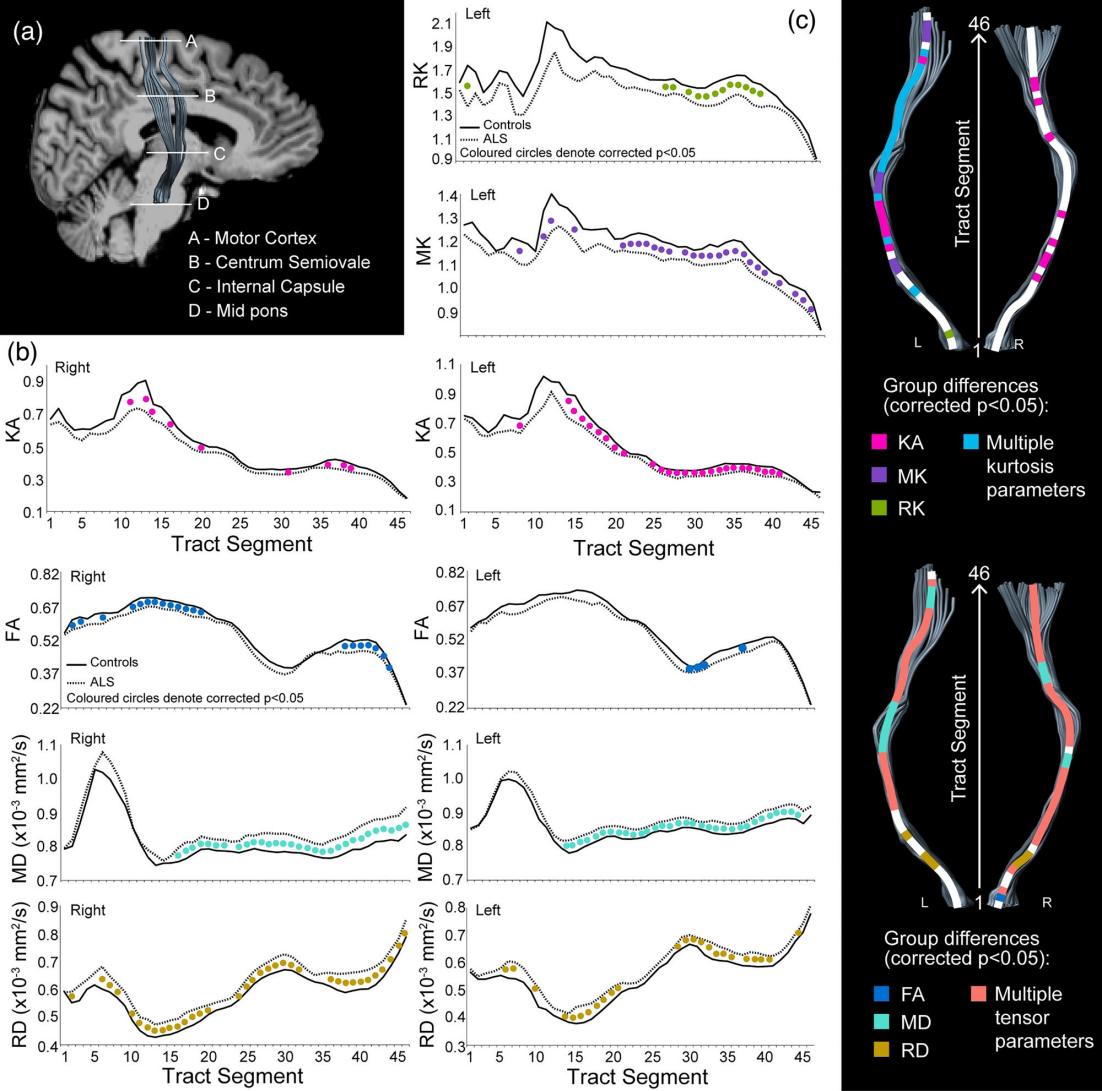
Along‐tract analysis of diffusion tensor and diffusion kurtosis metrics seen in the corticospinal tract in the patients in comparison to controls. (a) Anatomical subdivision of the corticospinal tract illustrated by superimposition of a sample tract extracted onto a T1 weighted image. (b and c) Changes observed along the right and left corticospinal tract in patient cohort in comparison to controls in diffusion tensor imaging (DTI) and diffusion kurtosis imaging (DKI) metrics, respectively. Solid circles along the tract represent significant group differences (*p* < .05, false‐discovery rate (FDR) corrected). The *X* axes represent position along the tract extending from the mid pons region to the motor cortex, and *Y* axes represents the tensor or the kurtosis metric measured.

**FIGURE 3:**
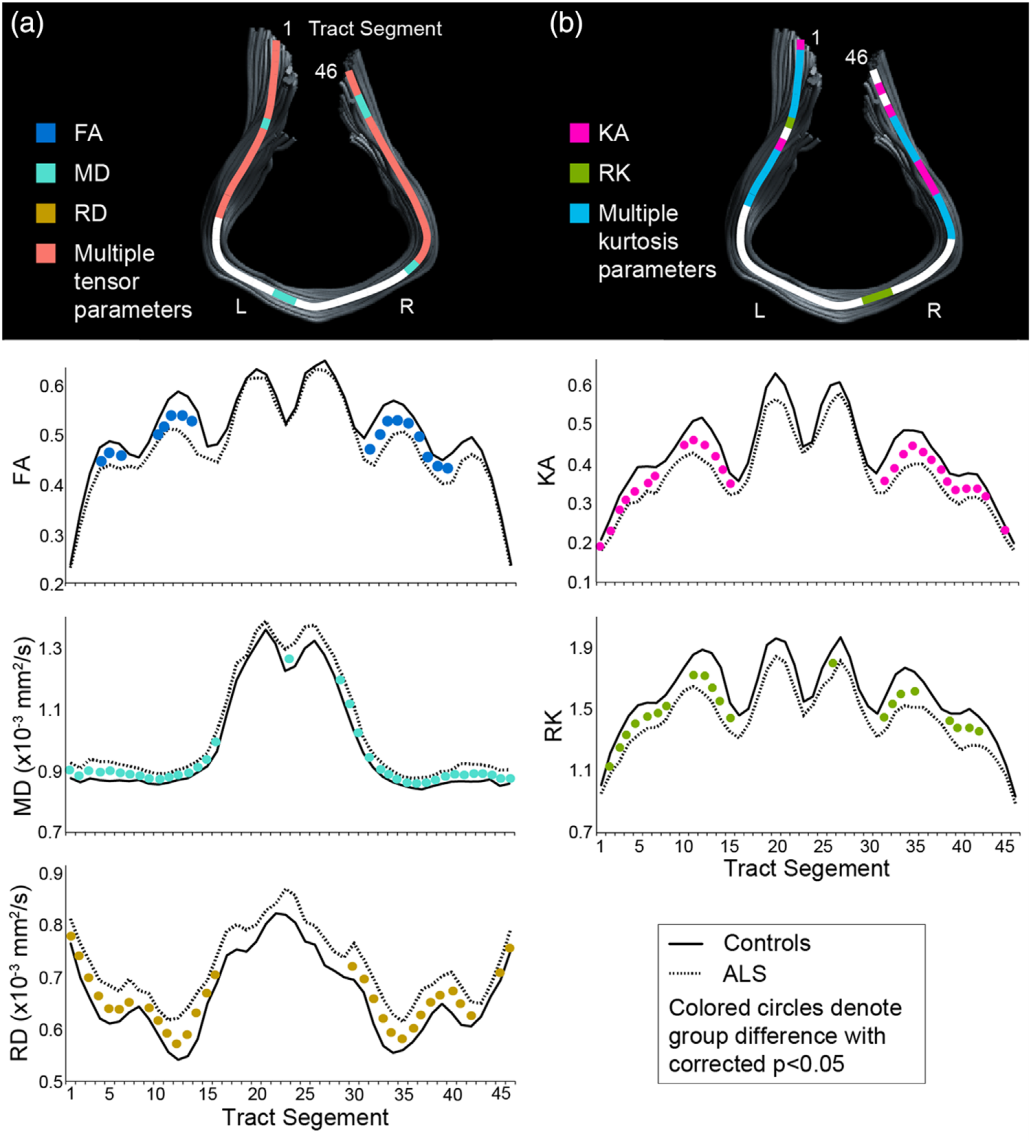
(a and b) Along‐tract differences observed along the motor fibers protruding from the body of the corpus callosum in patient cohort in comparison to controls in diffusion tensor imaging (DTI) and diffusion kurtosis imaging (DKI) metrics, respectively. Solid circles along the tract represent significant group differences (*p* < .05, false‐discovery rate (FDR) corrected). The *X* axes represent position along the tract extending from the mid pons region to the motor cortex, and *Y* axes represents the tensor or the kurtosis metric measured.

**FIGURE 4:**
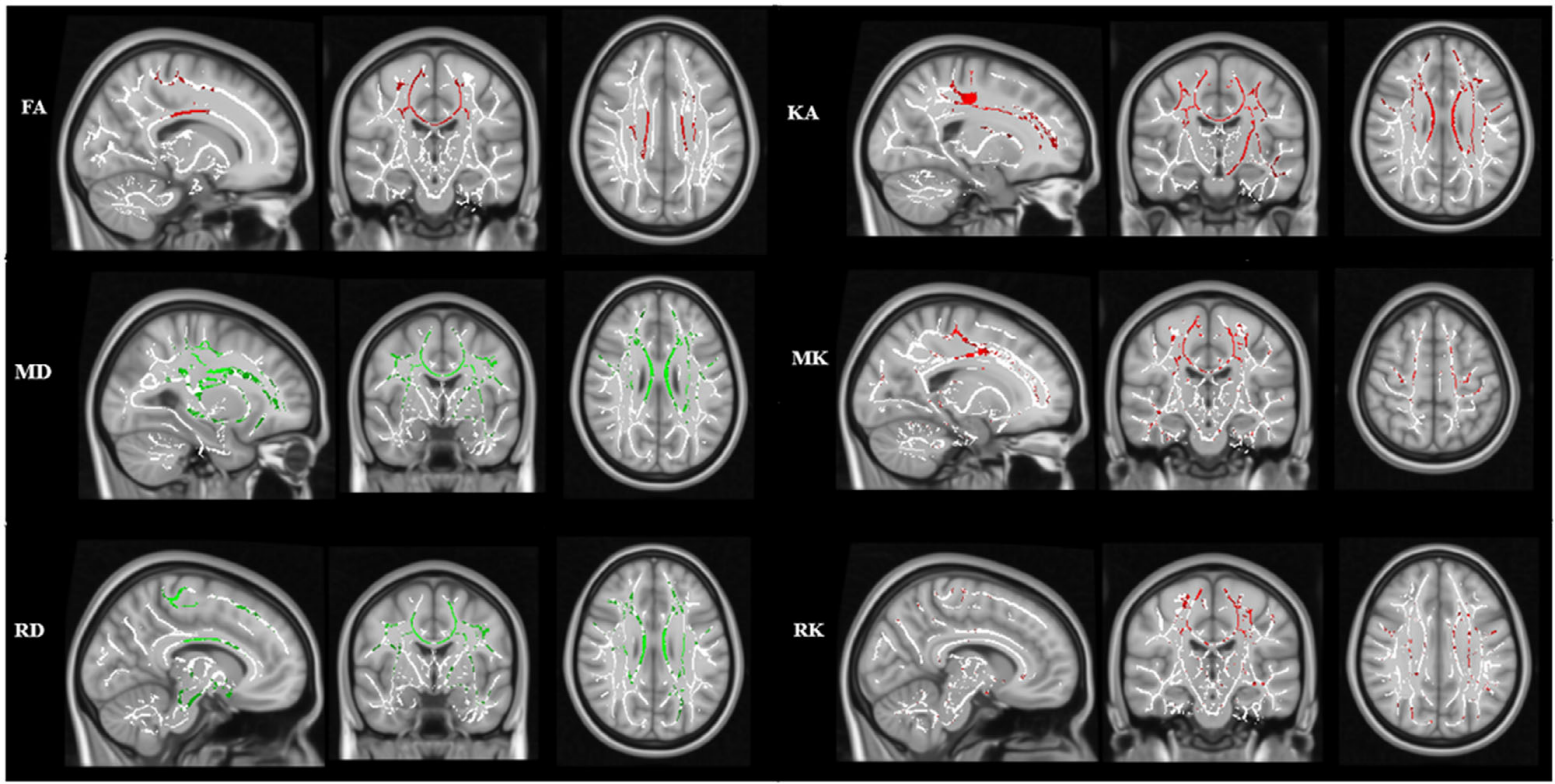
Tract‐based spatial statistics (TBSS) shows white matter (WM) regions with significant differences in diffusion tensor imaging (DTI) and diffusion kurtosis imaging (DKI) metrics between patients and healthy subjects (DTI, threshold‐free cluster enhancement [TFCE] corrected, *p* < .05; DKI, false‐discovery rate (FDR) corrected, *p* < .05). Red represents areas where parameter is significantly lower in patients; green represents areas where the parameter is significantly higher in patients.

### 3.5 TBSS DTI and DKI analysis (Figure 4)

TBSS was performed to corroborate along‐tract analysis results and to detect extra motor degeneration. TBSS analysis with DTI and DKI metrics revealed more widespread WM alterations in the patient cohort. Overall, DTI metrics (*p <* .05, TFCE corrected) and DKI (*p* < .05, FDR corrected) captured altered WM integrity in the bilateral CST and CC. Specifically, TBSS analysis revealed reduced FA and KA in the body of the CC and the bilateral CST. Patients displayed significant increase in MD in the body, splenium, and genu of the CC, bilateral CST, some frontal cerebral WM tracts; superior longitudinal fasciculus, forceps minor, anterior thalamic radiation, anterior corona radiata, and the anterior limb of the internal capsule in comparison to controls. Concurrently, lower MK was observed in the body of the CC, bilateral CST, superior longitudinal fasciculus, forceps minor, anterior thalamic radiation, and in the posterior limb of the internal capsule in patients relative to controls. Group comparisons revealed a higher RD predominantly in the bilateral CST, body of the CST, WM tracts of the cingulum, and the forceps minor in patients relative to controls. Similar pattern of WM alteration was noted by lower RK in patients as compared to controls. Lower MK and RK were observed in the fornix bundle of the limbic system in patients. No significant changes were observed in AD and AK metrics.

## 4 DISCUSSION

This study analyzed microstructural alterations in the bilateral CST and callosal motor fibers of patients with UMN dysfunction in comparison to controls. We sought to assess the ability of DKI metrics to detect the microstructural alterations in patients with UMN pathology. Our findings show that DKI metrics correlate with clinical signs of UMN degeneration and also revealed compromised tract integrity in the limbic circuitry. This suggests that DKI metrics such as MK and RK may complement diffusion analyses to reveal underlying pathology in UMN diseases.

DTI, although a robust noninvasive imaging tool and successful in detecting changes in WM integrity, may be limited in the degree to which it considers biological tissue complexity as it assumes Gaussian diffusion of water (Wu & Cheung, 2010). However, DKI accounts for the non‐Gaussian diffusion of water molecules occurring in biological tissues, making it an important imaging tool for the characterization of tissue microstructure in this respect. Previous findings suggest that microstructural alterations and compromised neuronal integrity arise from the glial activity and reactive astrogliosis resulting in a decrease in DKI metrics (Steven et al., 2014). Thus, DKI can potentially serve as a biomarker for neurodegenerative diseases and may be able to address the complexity of neural tissue beyond standard MRI imaging modalities.

Welton et al. (2019) reported decreased DKI measures in the motor cortex with concurrent increase in iron deposition in patients with mild‐to‐moderate ALS, indicating that DKI may be able to detect early pathological changes in ALS. In their voxel‐based DKI analysis, Huang et al. (2019) revealed reductions in MK and RK in both gray and WM regions in ALS patients. However, these studies do not address the use of DKI in assessing alterations of the major WM bundles affected in ALS and PLS, namely, the CST and motor fibers of the CC.

CST degeneration is considered the hallmark pathology in ALS. Consistent with the results of our study, previous DTI‐based tractography studies have observed consistently reduced FA and increased MD and RD values in the CST, particularly at the level of the internal capsule (Ishaque et al., 2019, Sarica et al., 2014, Sarica et al., 2017). Interestingly, a similar pattern of changes in DKI metrics was observed in our study along the CST that complement the results from previous DKI studies in ALS (Ciccarelli et al., 2009, Prudlo et al., 2012). Specifically, reduced KA, MK, and RK were observed in the WM tracts of the bilateral CST similar to previous DTI findings (Agosta et al., 2010, Ciccarelli et al., 2009, Prudlo et al., 2012, Sach et al., 2004). Additionally, significant correlations between contralateral UMN burden and MK of the CST were found, suggesting that these metrics reflect UMN degeneration leading to clinical dysfunction that was not present with DTI metrics. This suggests that DKI metrics might be more specific to CST/UMN pathology. Taken together, these findings in the CST suggest compromised microstructural integrity in the motor pathway, likely attributed to neuronal loss and altered WM pathology.

Previous studies have shown marked reductions of FA in the motor fibers from the body of the CC compared to the genu and splenium in patients with UMN disease (Cardenas et al., 2017, Chapman et al., 2014, Filippini et al., 2010). These results are congruent with our DTI and DKI findings, where lower KA, RK, and MK were also observed in the CC. Our TBSS findings suggest greater microstructural alterations occurring in the middle subregion of the CC, which is in line with a previous study in which decreased MK and RK values were observed in similar subregions of the CC (Huang et al., 2019). Another study also observed lower MK values in the CC genu and body in the early stages of ALS disease pathology in mice (Gatto et al., 2019). Our along‐tract analysis revealed the most pronounced WM alterations in the motor callosal fibers that projected into the motor cortex. Previous histopathological studies have suggested that reduction in FA in the body of the CC in ALS is not completely due to demyelination or axon degeneration, but rather a result of the aggregation of activated microglia or astrogliosis (Cardenas et al., 2017). Furthermore, these alterations to the motor callosal fibers of patients with UMN degeneration may also explain the altered neural network interactions observed in the patients (Bartels et al., 2008). Altogether, TBSS results also corroborated our findings at a group level and the along‐tract analysis, thereby pointing toward the potential use of DKI in detecting WM alterations in conjunction with DTI.

Interestingly, TBSS analyses also revealed reduced MK and RK values in the fornix of the limbic system, whereas no changes in DTI metrics were seen in this region. The fornix connects various nodes of the limbic system serving as a major output tract for the hippocampus, therefore playing an integral role in cognition and recall of episodic memory (Senova et al., 2020). Reduced MK is indicative of a loss of cellular structures; neuronal bodies, synapses, and dendrites, whereas reduced RK points toward compromised myelin and cellular membrane integrity (Steven et al., 2014). Several studies have reported compromised fornix and hippocampal structural integrity that can be associated with memory deficits and cognitive impairment observed in ALS patients (Christidi et al., 2019, Consonni et al., 2016, Kasper et al., 2015, Machts et al., 2018, Phukan et al., 2012). Therefore, our results show that MK and RK are probably uniquely capable of detecting microstructural alterations in complex crossing fibers as seen in the fornix, which is not evident using DTI alone. No significant changes in AD and AK values were observed in patients in the bilateral CST or CC as previously reported (Prudlo et al., 2012, Sarica et al., 2017). This could be because AD and AK are a measure of diffusion along the axial direction of axons and are relatively less constricted.

## 5 LIMITATIONS

The study has several limitations. First, cognitive function was not analyzed for subjects given the focus of the study on the motor system (CST and motor portion of the CC). Second, tractography was performed manually, and although repeated to ensure the accuracy of results, an automated approach will be more feasible and reproducible. Moreover, a relatively small sample size could result in decreased statistical power in detecting WM alterations in patients with UMN dysfunction. Additionally, TBSS findings reported for DKI survived only FDR correction; therefore, the efficacy of DKI metrics to detect microstructural alterations in extra motor regions observed requires careful interpretation. Future directions involve using a larger sample size in which tracts exhibiting crossing fibers can be analyzed using DKI, providing a more in‐depth understanding of disease pathology at a microstructural level.

## 6 CONCLUSION

Our findings suggest that the use of non‐Gaussian diffusion techniques such as DKI in conjunction with conventional DTI methods can provide a more in‐depth understanding of disease pathology in patients with UMN dysfunction. Additionally, our results probe the potential use of DKI metrics such as MK and RK as objective markers in detecting WM alterations in complex WM crossing fibers that might help in the early detection of disease for patients with UMN dysfunction.

## CONFLICT OF INTEREST STATEMENT

The authors declare no conflict of interest.

### PEER REVIEW

The peer review history for this article is available at https://publons.com/publon/10.1002/brb3.3102.

## Data Availability

The data that support the findings of this study are available on request. Contact Dr. Sanjay Kalra (kalra@ualberta.ca) for data requests.
